# Recommendations From a Chinese-Language Survey of Knowledge and Prevention of Skin Cancer Among Chinese Populations Internationally: Cross-sectional Questionnaire Study

**DOI:** 10.2196/37758

**Published:** 2023-03-09

**Authors:** Lily Ye Chen, Wei Niu, Kristina Lim, James A Solomon

**Affiliations:** 1 College of Medicine University of Central Florida Orlando, FL United States; 2 Health Sciences Center New Orleans Louisiana State University New Orleans, LA United States; 3 Division of Dermatology Lehigh Valley Health Network Allentown, PA United States; 4 College of Medicine Florida State University Tallahassee, FL United States; 5 Carle Illinois College of Medicine University of Illinois Urbana-Champaign Champaign, IL United States

**Keywords:** skin cancer, basal cell carcinoma, squamous cell carcinoma, melanoma, people of color, skin of color, risk and prevention, photoprotection, sun protection, sunscreen, prevention, cancer risk, cancer prevention, Chinese Asian, Chinese, Asian, North American Chinese, Fitzpatrick score, cultural difference, awareness, education

## Abstract

**Background:**

There is a paucity of studies assessing awareness and prevention of skin cancer among Chinese populations.

**Objective:**

The aim of the study is to compare attitudes and practices regarding skin cancer risks and prevention between Chinese Asian and North American Chinese populations and between Fitzpatrick scores.

**Methods:**

A cross-sectional, internet-based, 74-question survey in Chinese was conducted focusing on Han Chinese participants internationally. The survey included Likert-type scales and multiple-choice questions. All participants were required to read Chinese and self-identify as being 18 years or older and Chinese by ethnicity, nationality, or descent. Participants were recruited on the internet over a 6-month period from July 2017 through January 2018 via advertisements in Chinese on popular social media platforms: WeChat, QQ, Weibo, Facebook, and Twitter.

**Results:**

Of the 113 completed responses collected (participation rate of 65.7%), 95 (84.1%) were ethnically Han Chinese, of which 93 (96.9%) were born in China and 59 (62.1%) were female. The mean age of these 95 participants was 35.8 (SD 13.3) years; 72 (75.8%) participants were born after 1975. Few but more North American Chinese reported that Chinese Asian populations received annual skin checks (4/30, 4.2% vs 0/65, 0%; *P*=.009) and believed that their clinician provided adequate sun safety education (13/30, 43.3% vs 15/65, 23.1%; *P*=.04). Participants with higher Fitzpatrick scores less frequently received sun safety education from a clinician (4/34, 11.8% vs 22/61, 36.1%; *P*=.02). More participants with lower Fitzpatrick scores used sunscreen (41/61, 67.2% vs 16/34, 47.1%; *P*=.05), but alternative sun protection use rates are similar across groups.

**Conclusions:**

Cultural differences and Fitzpatrick scores can affect knowledge and practices with respect to sun protection and skin cancer among social media–using Chinese Asian and North American Chinese communities based on respondent demographics. Most participants in all groups understood that people of color have some risk of skin cancer, but >30% of all groups across regions and Fitzpatrick scores are unaware of current skin protection recommendations, receive insufficient sun safety education, and do not use sunscreen. Outreach efforts may begin broadly with concerted public and private efforts to train and fund dermatologists to perform annual total body skin exams and provide more patient education. They should spark community interest through mass media and empower Chinese people to perform self-examinations and recognize risks and risk mitigation methods.

## Introduction

Skin cancer is a global health issue. Among people of color (POC), the outcomes are far worse than that of the general population [1-8]. This poorer outcome has been associated with delay in diagnosis due to the failure of the medical community and patients to recognize the distinct risks [4,5,9-11] and signs [4,11-15] of skin cancer in non-White populations.

China is the most populous country with approximately 1.4 billion people; Han Chinese is the largest ethnic group native to China, making up 92% of the Chinese population and 19% of the global population. Despite the attention to skin in the market for skin products and treatments among Chinese consumers, skin cancer incidence rates continue to increase in Chinese populations [14,16-23].

One study noted that, between 2004 and 2011, the overall incidence of melanoma in China increased from 0.4/100,000 to 0.48/100,000 [21]. A study of long-term trends of skin malignant melanoma in China between 1990 and 2019 reported annual incidence net drifts of 3.523% and 3.779% for males and females, respectively [23]. In tandem with China’s rapidly aging population, the age-standardized incidence rate of melanoma in China has increased as well [23], increasing by 110.3% from 1990 to 0.9/100,000 in 2017 [17]. Among the Chinese population of Hong Kong, between 1990 and 1999, the incidence of basal cell carcinoma increased from 0.32/100,000 to 0.92/100,000, and the incidence of squamous cell carcinoma from 0.16/100,000 to 0.34/100,000 [14]. In Singapore, between 1968 and 2016, the age-standardized incidence rate of cutaneous basal cell carcinoma among ethnically Chinese people increased from 2.7/100,000 to 6.9/100,000 [22].

Nonetheless, compared to the wealth of research on skin cancer risks, incidence, awareness, and prevention among Western White and people of color populations [24-29], there is a paucity of publications concerning that of Chinese populations. A recent review paper reported the identification of only 9 papers across numerous Western and Chinese English-accessible databases that studied knowledge, attitudes, beliefs, and behaviors related to skin cancer and sun protection in China [30]. In Chinese, a limited collection of peer-reviewed and non–peer-reviewed research has been published in the past decade [31-37]. Chinese people internationally are often [8,10,28,38]—but not always [16,27,39]—grouped into Asian and East Asian categories for studies related to skin cancer rates and behavioral risks, impeding extrapolation for subgroups. Only 2 studies were identified comparing the perspectives on the risks of skin cancer among specific ethnic Chinese populations from different sociocultural backgrounds [38,40].

In this study, international Han Chinese perspectives on skin cancer were recruited through social media and anonymously surveyed in simplified Chinese. Trends and gaps in knowledge of risk factors and preventative measures were identified and used to determine necessary educational measures for developing future interventions with patients, educators, and providers.

## Methods

### Participant Recruitment

Participants were recruited on the internet over a 6-month period via advertisements in Chinese on popular social media platforms: WeChat (Tencent Holdings Limited), QQ (Shenzhen Tencent Computer System Co, Ltd), Weibo (Sina Corporation), Facebook (Meta Platforms, Inc), and Twitter (Twitter, Inc). All participants were required to read Chinese and self-identify as being 18 years or older and Chinese by ethnicity, nationality, or descent. No financial compensation was provided.

### Survey Details

An internet-based survey was adapted into Chinese from an English survey used in previous studies for assessing current knowledge and management of skin cancer in English-speaking populations [41-44]. The survey contained 74 questions in Chinese (Multimedia Appendix 1) and took approximately 30 minutes to complete. The University of Central Florida Institutional Review Board approved the Chinese-adapted version. Participants completed the survey on SurveyMonkey cloud-based software (SVMK Inc). Survey contents and results are reported in English.

### Data Analysis and Visualization

Data analysis and visualization were completed in Excel (Microsoft Corporation) and R (R Foundation for Statistical Computing). Comparisons with chi-square and Fisher exact tests were made—for query responses with all counts 10 or greater and at least 1 less than 10—between responses by Chinese participants in Asia (group 1) versus those of Chinese participants in North America (group 2), and by those with modified Fitzpatrick scores ≤14 (modified Fitzpatrick group 1 [FG1]) versus those with modified Fitzpatrick scores ≥15 (modified Fitzpatrick group 2 [FG2]).

Due to the relative homogeneity in ethnicity, hair color, and eye color among ethnically Chinese people, scores were determined as a summation of points from questions modified to be more specific to skin phototyping for Chinese skin types [45] and more granular than the conventional Fitzpatrick scoring system:

1. How dark is your skin normally? (0=albino; 7=very dark brown)
2. How dark do you get if you tan? (0=albino; 7=very dark brown)
3. How easy is it for your skin to tan? (0=never tans; 7=always tans)
4. How easy is it for your skin to sunburn? (0=always sunburns; 7=never sunburns)

Since the maximum possible score was 28, half of the maximum (14) was chosen to be the dividing value between FG1 and FG2. In short, participants in FG1 had paler skin or experienced more sensitivity to burning than their FG2 counterparts.

### Ethical Considerations

This study has been reviewed and exempted by UCF institutional review board (IRB No. SBE-17-12900).

## Results

The response rate was 113 of 172 (65.7%). Of this subset, 95 (84%) participants were of Han Chinese ethnicity. Only the results from fully completed surveys by Han Chinese participants are reported to reduce cross-cultural confounding factors. Participant demographics and history of skin cancer are summarized in Table 1. Of note, 93 (98%) of respondents were born in China, while 64 (67%) currently live in China. Comparisons between responses from different geographical locations are shown in Table 2. A modified Fitzpatrick scale was used to determine whether the variable darkness of the skin among Chinese contributes to skin cancer risks and perception in Table 3. Among all participants, 37 (39%) reported not using any sunscreen; the primary cited reasons for not doing so are summarized in Table 4. The use of alternatives options to sunscreen are shown in Table 5 (participants could select more than one option as listed).

**Table 1:** Participant demographics and history of skin cancer (n=95).

Characteristic		Participants, n (%)
**Han Chinese ethnicity**		95 (100)
	College or higher degree	66 (70)
	Born in China	93 (98)
	Born after 1975, residence in Asia	54 (57)
	Born after 1975, residence in North America	18 (19)
**Geographical region of residence**		
	China	64 (67)
	Other Asian countries	1 (1)
	United States	26 (27)
	Canada	3 (3)
	Australia	1 (1)
	Other countries (unspecified)	1 (1)
History of precancerous and cancerous lesions		1 (1)

**Table 2:** Skin cancer knowledge and preventative measures between regions. Survey responses from Han Chinese are displayed, and responses are separated for comparison into 2 groups based on the participants’ region of residence: Asia (group 1) and North America (group 2). The sample sizes from other geographical regions were too small for statistical analysis.

Category			Han, n (%)		Group 1, n (%)		Group 2, n (%)		*P* value	
Group size			95 (100)		65 (100)		30 (100)		N/A^a^	
Skin cancer risk in Chinese less than White people			73 (77)		46 (71)		27 (90)		.06	
No skin cancer risk in Chinese			10 (11)		9 (14)		1 (3)		.16	
POC^b^ can get skin cancer			86 (91)		58 (89)		28 (93)		.78	
Can identify melanoma as a type of skin cancer			57 (60)		42 (65)		15 (50)		.18	
Have not read about the latest skin care recommendations			82 (86)		56 (86)		26 (87)		>.99	
Interested but do not know how to get resources for recommendations			35 (37)		27 (42)		8 (27)		.18	
Know that sunscreen protects from skin cancer			65 (68)		42 (65)		23 (77)		.34	
**Level of concern about skin cancer in lifetime**										.83
	Do not worry	39 (41)		26 (40)		13 (43)				
	Mild to moderate	50 (53)		34 (52)		16 (53)				
	Serious	6 (6)		5 (8)		1 (3)				
**Likelihood of seeing clinician for new skin lesion**										.84
	Never	8 (8)		5 (8)		3 (10)				
	Unlikely	29 (31)		21 (32)		8 (27)				
	Likely to very likely	58 (61)		39 (60)		19 (63)				
Clinician discussed risks of tanning			2 (2)		1 (2)		1 (3)		.53	
Clinician discussed skin cancer risks			6 (6)		4 (6)		2 (7)		>.99	
Received annual skin check			4 (4)		0 (0)		4 (13)		.009	
Received sun safety education from clinician			26 (27)		19 (29)		7 (23)		.63	
Felt clinician gave adequate sun safety education			28 (30)		15 (23)		13 (43)		.04	
Sunbathe to tan			6 (6)		2 (3)		4 (13)		.08	
Uses sunbeds or tanning booths			0 (0)		0 (0)		0 (0)		>.99	

^a^N/A: not applicable.

^b^POC: people of color.

**Table 3:** Skin cancer knowledge and preventative measures between Fitzpatrick groups. Survey responses from Han Chinese are separated for comparison into 2 groups based on the participants’ Fitzpatrick scores: those with modified Fitzpatrick score ≤14 (modified Fitzpatrick group 1 [FG1]) and those with modified Fitzpatrick score ≥15 (modified Fitzpatrick group 2 [FG2]).

Category			FG1, n (%)		FG2, n (%)		*P* value	
Group size			61 (100)		34 (100)		N/A^a^	
Skin cancer risk in Chinese less than White people			48 (79)		25 (74)		.62	
No cancer risk in Chinese			5 (8)		5 (15)		.49	
POC^b^ can get skin cancer			55 (90)		31 (91)		>.99	
Can identify melanoma as a type of skin cancer			37 (61)		20 (59)		.86	
Have not read about the latest skin care recommendations			52 (85)		30 (88)		.77	
Interested but do not know how to get resources for recommendations			24 (39)		11 (32)		.50	
Know that sunscreen protects from skin cancer			47 (77)		18 (53)		.02	
**Level of concern about skin cancer in lifetime**								.75
	Do not worry	23 (38)		16 (47)				
	Mild to moderate	34 (56)		16 (47)				
	Serious	4 (7)		2 (6)				
**Likelihood of seeing clinician for new skin lesion**								.30
	Never	3 (5)		5 (15)				
	Unlikely	19 (31)		10 (29)				
	Likely to very likely	39 (64)		19 (56)				
Clinician discussed risks of tanning			2 (3)		0 (0)		.32	
Clinician discussed skin cancer risks			5 (8)		1 (3)		.54	
Received annual skin check			3 (5)		1 (3)		>.99	
Received sun safety education from clinician			22 (36)		4 (12)		.02	
Felt clinician gave adequate sun safety education			18 (30)		10 (29)		.99	
Sunbathe to tan			4 (7)		1 (3)		.65	
Uses sunbed or tanning booth			0 (0)		0 (0)		>.99	

^a^N/A: not applicable.

^b^POC: people of color.

**Table 4:** Reasons for not using sunscreen. The proportion of respondents who denied using sunscreen and their cited reasons are tabulated. Participants were only able to select one reason for not using sunscreen. *P* values were calculated for Asia (Group 1) versus North America (Group 2) with *P* value A and Fitzpatrick score ≤14 (modified Fitzpatrick group 1 [FG1]) versus Fitzpatrick score ≥15 (modified Fitzpatrick group 2 [FG2]) with *P* value B.

Category			Han (n=95), n (%)		Group 1 (n=65), n (%)		Group 2 (n=30), n (%)		*P* value A		FG1 (n=61), n (%)		FG2 (n=34), n (%)		*P* value B
Do not use sunscreen			37 (39)		27 (42)		10 (33)		.45		20 (33)		18 (53)		.05
**No need to use sunscreen^a^**			10 (27)		6 (22)		4 (40)		.83		7 (35)		3 (17)		.24
	Inconvenient to use sunscreen	11 (30)		8 (30)		3 (30)				3 (15)		8 (44)			
	Choose other means of sun protection	2 (5)		2 (7)		0 (0)				1 (5)		1 (6)			
	Do not know how to use sunscreen correctly	1 (3)		1 (4)		0 (0)				1 (5)		0 (0)			
	Other reasons	13 (35)		10 (37)		3 (30)				8 (40)		6 (33)			

^a^Percentages in this category are based on the “do not use sunscreen” numbers.

**Table 5:** Sun protection aside from sunscreen. Methods other than sunscreen used to protect against UV exposure outdoors are detailed. Some respondents have overlaps between categories as well as with sunscreen usage. *P* values were calculated for group 1 versus group 2 (*P* value A) and FG1^a^ versus FG2^b^ (*P* value B).

Category	Han (n=95), n (%)	Group 1 (n=65), n (%)	Group 2 (n=30), n (%)	*P* value A	FG1 (n=61), n (%)	FG2 (n=34), n (%)	*P* value B
Wide-brimmed hats or long-sleeve clothing	58 (61)	39 (60)	19 (63)	.76	40 (66)	18 (53)	.23
Sunglasses	59 (62)	35 (54)	24 (80)	.02	36 (59)	23 (68)	.41
Umbrella	48 (51)	38 (59)	10 (33)	.02	34 (56)	14 (41)	.17

^a^FG1: modified Fitzpatrick group 1.

^b^FG2: modified Fitzpatrick group 2.

## Discussion

### Overview

Skin lightening is a multibillion dollar industry among Chinese people. Despite Chinese culture’s well-known and generally strong preferences for whiter, lighter-toned skin [46-48], limited research has been done on Chinese knowledge and practices with respect to sun protection and skin cancer.

Given that skin cancer incidence rates and mortality continue to increase among Chinese people [14,16-19,49], it is imperative to understand and identify optimal strategies to synergize with consumer interests for effective UV radiation protection. This study is the first to compare Chinese attitudes and practices between Chinese Asian and North American Chinese populations as well as between modified Fitzpatrick scores.

In this study, most participants were Han Chinese, which is consistent with Chinese ethnic demographics. Most participants were born in China after 1975 (Table 1), the year when a generational paradigm shift was instituted, altering from traditional to modern Chinese culture and economics. Thus, our findings and recommendations are more focused on the perspectives and knowledge of younger generations who are more highly educated and actively use and were recruited through social media.

Consistent with the participants’ age distribution, only one of the participants had a history of precancerous or cancerous lesions (Table 1). The incidence rate of skin cancer increases with age across races [2,18,50,51], and Chinese patients are more likely to be diagnosed with skin cancer after 40-60 years of age [17,52].

### On the Risks Factors of Race and Ethnicity

While participants across all groups predominantly (>89%) believe that POC can get skin cancer, most participants believe that Chinese people are less at risk than Caucasian people (Tables 2 and 3). North American Chinese people may believe more often than their Chinese-Asian counterparts in a lower skin cancer risk (*P*=.06, Table 2). However, there is no significant difference between FG1 and FG2.

The potential significance of geographic location could be linked to experience bias. The higher awareness of skin cancer by Chinese people in North America may be related to living in heterogenous communities, wherein non-Asian counterparts are subject to skin cancer. Nonetheless, there is consistent recognition across regions and Fitzgerald scores that skin color does not guarantee immunity to skin cancer.

### On Knowledge of Melanoma and Skin Care

Knowledge about skin cancer is limited in Chinese communities; only 50.0%-64.4% of participants can define melanoma as a type of skin cancer (Tables 2 and 3). Neither modified Fitzpatrick score nor geographical location yielded statistically significant differences in this lack of knowledge.

Group 1 and group 2 have read the latest skin care recommendations at comparable rates (*P*>.99, Table 2). Acquiring knowledge of skin cancer risk may be more associated with interest and motivation as opposed to resource access (Tables 2 and 3).

### On Interest and Clinical Care Related to Skin Cancer

Across location and modified Fitzpatrick score groups, 37.7%-47.1% of respondents in each group lacked concern regarding the risk of skin cancer in their lifetime (Tables 2 and 3).

While most participants are either likely or very likely to see a clinician for a new lesion, participants consistently reported low rates of annual skin checks; significantly more annual skin checks occured in North America than in Asia (*P*=.009, Table 2). This rate does not appear to be affected by the modified Fitzpatrick score (Table 3).

Most Chinese people across all groups had neither received sun safety education from their clinicians nor were generally satisfied with the education when provided (Tables 2 and 3). However, within these findings, significantly more North American Chinese people felt satisfied with the education provided (*P*=.04, Table 2). Furthermore, significantly more participants in FG1 than in FG2 received sun safety education from clinicians (*P*=.02, Table 3).

### On Tanning Practices

While no participants used sunbeds, outdoor sun tanning practices were more popular among North American Chinese than Chinese Asian people (*P*=.08, Table 2), which would be in agreement with the existing literature [38]. Consequently, Western clinicians should recognize this behavior and be proactive in initiating sun safety discussions with ethnically Chinese people living in North America. In Asia, the monitoring of trends should continue, and dedicated educational programs on sunbathing and tanning should be proactively implemented.

### On Sun Protection Practices

Sunscreen use was reported in 47.1%-67.2% of participants across locations and modified Fitzpatrick score groups (Tables 2-4). Participants in FG1 may use sunscreen more frequently than their counterparts in FG2 (*P*=.05, Table 4).

Nonetheless, a sizeable minority do not use sunscreen. Efforts are needed to confirm that these individuals are using other forms of UV protection, including hats, umbrellas, and sunglasses; in this study, 35 (37%) of all 95 Han Chinese participants stated that they used no forms of UV protection at all (Table 4). It is furthermore pertinent to conduct additional surveys to confirm that sunscreen is being applied at appropriate time intervals and in appropriate volumes.

No significant differences concerning the lack of sunscreen use were found between group 1 and group 2 nor FG1 and FG2 (Table 4), suggesting similar viewpoints between groups.

It is worthwhile to note that some people reported a lack of knowledge on correct sunscreen use (Table 4). Perhaps sunscreen manufacturers could add to their products QR codes linked to instructional videos for proper sunscreen applications [53].

In terms of alternative methods for sun protection, group 1 and group 2 used wide-brimmed hats and long-sleeve clothing at similarly high rates (39/65, 60% vs 19/30, 63%, Table 5). Use of sunglasses for sun protection had a much higher proportion (35/65, 54% vs 24/30, 80%) in group 2 than in group 1 (*P*=.02, Table 5). This statistically significant difference is understandable due to the popularity of sunglasses in North American 20th century culture [54]. Although sunglasses are growing in popularity, their use remains minimal for sun protection in Asia [30,55-57]. On the other hand, “sun umbrellas” use among Chinese-Asian people, especially Chinese-Asian women, is frequently used to maintain a white complexion [30,55-58].

Between FG1 and FG2, protective clothing, sunglasses, and umbrella use rates were similar between the groups (Table 5). As such, modified Fitzpatrick scores are less likely to affect sun protection practices beyond sunscreen.

### Closing the Educational Gap

It is imperative to educate and motivate Chinese communities to intervene in the growing severity of diagnoses and incidence of skin cancer. Given the similarities in responses between groups, it is not unreasonable to begin with a standard guide translated into various languages and methodology for addressing skin cancer knowledge and behavior between clinicians and Chinese patients in various languages. Effective dissemination of educational messages can be achieved via social media and other forms of mass media [30,59]. Additional research should be conducted to identify viewpoints shared among participants and develop effective media-based outreach for skin cancer prevention campaigns, which may be accomplished using a method like that of Shi et al [60].

Moreover, Chinese communities have expressed interest in skin exams and increased breadth and depth of sun safety education. Efforts should be made in dermatology residency programs internationally to emphasize skin cancer risks, signs, and symptoms among all skin types, including Chinese; review specific techniques for skin protection to aid in patient education; and train residents to complete total body skin exams (TBSEs). We recommend that annual TBSEs should be conducted by a dermatologist.

Current screening guidelines confound this recommendation—the US Preventative Services Task Force states that there is insufficient evidence to determine the effectiveness of visual skin exam screenings in US patients without obvious related signs or symptoms [61]; however, the methods behind this recommendation have been extensively critiqued [62], and notably, the conclusions are based on data inclusive of primary care clinicians alongside dermatologists without a direct means of comparison between screening accuracy [61]. Organizations’ recommendations for other regions vary from no recommendations to self-examination twice a year for specific high-risk populations to 2-year intervals for all individuals from the age of 35 years onward [63,64]. Nevertheless, emerging data demonstrate that TBSEs conducted by dermatologists are low-cost and efficacious as a screening tool for detecting skin cancer [65], and they detect skin cancer at significantly higher rates than partial skin exams; for malignant melanoma, it is suggested that a dermatologist-conducted TBSE is 23.5 times more likely to identify a lesion than a Pap smear to identify a cervical cancer lesion [66].

Differences in health care systems provide another challenge to implementing TBSEs. In China, traditional Chinese medicine (TCM) is practiced alongside Western medicine, each with its own set of diagnostics, interpretations, therapeutics principles, and treatments [67,68]. Different logic systems are in place, including for cancer, with some analogous language and principles [69]. Both forms of medicine recognize the value of preventative medicine through early detection and treatment [67-69]; this shared perception should be used to adequately reach out to Chinese communities in China and abroad that preferentially rely on TCM. Collaboration with TCM universities to reconcile and integrate knowledge related to skin cancer risks, prevention, and screening into their curriculum and improve the cultural competency of allopathic clinicians to provide parallels to TCM concepts will improve the care and patient education of TCM patients [68,70-74].

Furthermore, China’s multitiered health care system is intended to coordinate between primary health care with general practitioners and secondary and tertiary health care at hospitals, with more complex levels of care and with more resources available at higher-tiered hospitals. Currently, there is limited use of primary health care services in China and preferential use of hospitals for medical services [75]. The type of first-visit hospitals and socioeconomic status have also been shown to significantly impact the time for diagnosis of melanoma [76]. In addition to ongoing reform efforts, one method to address these limitations would be to fund annual TBSEs by dermatologists as a part of routine primary care, which would lessen the financial burden and incentivize more patients to receive timely screenings.

Supplementarily, self-examination techniques should be taught through private and public health organizations to be conducted at regular intervals appropriate to individuals’ genotypic, phenotypic, and environmental risk levels; all communities should be encouraged to seek clinical evaluation for lesions identified by tools such as the ABCDE rule (asymmetry, border irregularity, color nonuniformity, diameter >6 mm, and evolution) or the “ugly duckling” sign. Resources for POC to recognize their risks of malignancy and methods to protect against UV radiation—such as how to properly apply sunscreen—should be commonplace, and some examples can be found on the American Academy of Dermatology [77], American Cancer Society [78], and other organization websites. *Mind the Gap*, an extensive open-source handbook, compiles clinical signs in black and brown skin [79], and such efforts further aid in broadening the understanding and awareness among patients, educators, and clinicians. A selection of sun safety and skin cancer–related resources are available within China from government and nongovernment sources, some of which are highlighted in Multimedia Appendix 2 [80].

For the individual patient, culture, phenotypes, and lifestyles can significantly influence responses to upon all steps of the process, from information intake to application. Thus, all these factors ultimately should be considered in individualized educational programs and clinicians’ care for Chinese patients both in Asia and in North America.

### Limitations and Future Directions

Aside from the limitations of recall bias for survey-based research, future comparisons of groups by demographics of sex, age, and level of education would elucidate further stratifications of attitudes and practices and may provide suggestions for tailoring educational programs more specifically for individual patients [19,30,55,81]. While these demographic data were collected, distributions were insufficient to make meaningful inferences, except that our findings and recommendations from this study are primarily directed toward Chinese populations that use Chinese-language social media. At the time of surveying, Chinese-Asian participants on average were 32.1 years of age, 7.5 years younger than their North American Chinese participants at 40.6 years of age. Although this age difference is a limitation of our study, individuals of these groups are not void of skin cancer risks. Future studies with other languages, as discussed below, will better encapsulate younger populations.

Contextual exposure to UV radiation was not accounted for as part of this study. Though it certainly influences practices for sun protection and the risk of skin cancer, everyone has noninsignificant exposure risks to UV radiation. Among melanoma cases, the most common subtypes in China are acral and mucosal, followed by superficial spreading [6,64,82]. While UV radiation damage is primarily identified as the etiology of superficial spreading and has only been associated with a subset of mucosal and acral melanomas [82-84], reduction of risk against UV-induced damage overall would relieve the disease burden of skin cancer among the large population of Chinese people worldwide.

We consequently plan to expand our survey questions and recruit more participants to gain further insight concerning awareness of, exposure to, and behavior related to vocational and avocational exposure risks to UV radiation effects on skin health [36,37,85-88]. We then plan to develop community-specific best-practice recommendations adapted from existing methods [89] to mitigate these exposure risks. These include occupational and public health policies for communication, training, and protective equipment, and encourage use of the materials by making them conveniently accessible based on survey responses. Depending on the responses, for various situations, we will recommend different interventions such as onboarding training, protective clothing appropriate to the climate and conditions, shade structures, and sunscreen dispensers placed in locations frequented by workers in a certain industry; based upon the findings of Walkosz et al [89], these types of interventions likely will reduce the incidence of sun damage. Regular targeted free awareness-raising and screening events following the structure detailed by programs such as the American Academy of Dermatology “SPOT Skin Cancer” initiative [90,91] would also benefit populations associated with an identified high risk of skin cancer.

Given the population size of Han Chinese and the diaspora across the globe, new surveys will capture additional demographics and clarify regional geographical differences in cancer incidence and burdens within different Chinese provinces [17,92]. Maintaining the criteria of Chinese ethnicity while including different translations of the survey would provide better insight into the effects of geographical and cross-cultural differences. Thus, surveys should be available in both traditional and simplified Chinese, as well as languages of countries that currently have the largest overseas Chinese populations, including but not limited to English, Russian, Spanish, French, Italian, Indonesian, Thai, and Malay [93].

Furthermore, we will collaborate with more Chinese dermatology researchers and clinicians to expand our outreach. The collaboration would facilitate the surveying of more older participants who were born prior to 1975, allowing us to compare viewpoints between generations.

### Conclusions

In conclusion, our Chinese-language survey was used to assess and compare Han Chinese attitudes and practices related to skin cancer risks and prevention. We identified manifestations of cultural differences between Chinese Asian and North American Chinese communities that use social media, and we determined that opinions and behaviors among Han Chinese people may differ by modified Fitzpatrick score.

From our findings, we proposed several aims for educational programs by clinicians and health care organizations in Asia and North America for the largest ethnic group in the world. Through a collective and adaptive effort across all levels of health care, knowledge and practices with respect to sun protection and skin cancer among Chinese populations globally can be improved to reduce morbidity and mortality among this subset of POC.

## Appendix

Multimedia Appendix 1 Survey questions in Chinese as seen by participants and English translation of questions.

Multimedia Appendix 2 Select highlights of sun safety and skin cancer–related resources from China.

## Abbreviations

FG1: modified Fitzpatrick group 1
FG2: modified Fitzpatrick group 2
POC: people of color
TBSE: total body skin exam
TCM: traditional Chinese medicine
