# Supplementary material for: Recommendations From a Chinese-Language Survey of Knowledge and Prevention of Skin Cancer Among Chinese Populations Internationally: Cross-sectional Questionnaire Study
Source: JMIR Dermatol. 2023 Mar 9;6:e37758. doi: 10.2196/37758 (PMC10335128; doi:10.2196/37758)
Supplement: Multimedia Appendix 1 [file derma_v6i1e37758_app1.docx]

**Please note that the English translation begins on page 18 of this document**

研究题目: 研究华人群体对皮肤癌基本知识和治疗的认知

主要研究人：[编辑出版]

我们邀请你参与这一研究。你可以自己决定是否参与。

皮肤癌是个困扰全球的医学疾病。在美国，每小时就有一位皮肤癌包括黑色素瘤，基底细胞癌和鳞状细胞癌患者死亡。其中非白人的有色人种在诊断为皮肤癌后的临床预后更差。其原因可能是医学界和病人本人都没有能早期充分评估这一疾病的风险，从而耽误了诊断。尽管在不同有色人种患病率并不一致，但在美国有超过200万有色人种有患皮肤癌危险。华人是全球人口最多的一个族群。相关研究表明皮肤癌发病率在华人群体逐渐增高。不过， 相对于美国白人族群，华人群体皮肤癌相关风险的研究还不够充分，因此有待于进一步针对做全球华人，包括居住在美国，中国以及全球其它地域华人对皮肤癌风险性等相关基本认知的研究。作为研究的参与者，你既可以是皮肤癌或者皮肤癌前病变的患者，也可以是没有皮肤癌的健康者。即使作为没有皮肤癌的健康者，你对皮肤癌的相关认识也有助于我们评估全球华人群体对这一疾病风险性和预防措施的认知是否全面。

本研究目的是调查全球华人在皮肤癌风险以及预防措施方面是否存在有相关认知的不足，这将有助于今后弥补这些不足。

你将填写一个耗时30分钟左右的匿名调查表。你可以在任何时间和有任何有网络连接的地方完成这一调查表。

你必须年满18岁才有资格完成这一调查。

如果你对研究有任何问题可以联系： [编辑出版]

IRB 机构可以保障你在研究中的权利和举报任何不足之处。中佛罗里达大学的和人相关的研究受到大学研究评估委员会的监督([编辑出版] IRB)。这项研究经该委员会评估并批准。如果想知道研究参与者享有哪些权利，请联系[编辑出版].

1. 你已经年满18岁, 能阅读中文，并且同意参与这项研究吗？

是

否

1. 你的性别？

男

女

1. 你的出生年代？
2. 你的出生国？

中国

其它任何亚洲国家

美国

加拿大

欧洲

澳大利亚

太平洋岛国

其它国家

1. 你现在居住国？

中国

其它任何亚洲国家

美国

加拿大

欧洲

澳大利亚

太平洋岛国

其它国家

1. 你是华人或者有华人血统吗？

是

否

1. 如果你在前一个问题回答“是”，请填写你的民族

汉族

蒙古族

回族

藏族

维吾尔族

苗族

彝族

壮族

布依族

朝鲜族

满族

侗族

瑶族

白族

土家族

哈尼族

哈萨克族

傣族

黎族

其它族

1. 你在中国出生吗？

是

否

1. 如果你中国出生，请选择你的祖籍？

安徽

北京

重庆

福建

甘肃

广东

广西

贵州

海南

河北

黑龙江

河南

湖北

湖南

江苏

江西

吉林

辽宁

内蒙古

宁夏

青海

陕西

山东

山西

上海

四川

台湾

天津

西藏

新疆

云南

浙江

香港

澳门

1. 你最高教育程度？

未受教育

小学

初中

高中

大学

研究生

11．你的职业？（如果是学生，选择学生。如果无业，选择无业）

无业

农业

会计

广告

航天

空运

服装及配饰

汽车

银行

生物技术

广播

客服中心

货运

化学

计算机

建筑

消费者产品

化妆品

教育

电子

能量

娱乐和休闲

金融服务

食品，饮料和烟草

政府

杂货

保健

网络印刷

司法

制造

电影音像

音乐

报纸印刷

拍卖

制药

出版

地产

零售批发

服务业

软件

学生

运动

技术

通信

电视

运输

卡车

军队

其它（请描述）

12.如果你有第二职业，请选择（如果是学生，选择学生；如果没有第二职业，选择无第二职业）

无业

农业

会计

广告

航天

空运

服装及配饰

汽车

银行

生物技术

广播

客服中心

货运

化学

计算机

建筑

消费者产品

化妆品

教育

电子

能量

娱乐和休闲

金融服务

食品，饮料和烟草

政府

杂货

保健

网络印刷

司法

制造

电影音像

音乐

报纸印刷

拍卖

制药

出版

地产

零售批发

服务业

软件

学生

运动

技术

通信

电视

运输

卡车

军队

其它（请描述）

13. 你的大部分工作主要在什么场所？

室内

室外

一半室内，一半室外

14. 你被诊断过皮肤癌前病变或者皮肤癌吗？

是

否

15. 如果你在前一个问题回答“是”，你被诊断过哪种皮肤癌前病变或者皮肤癌？（可以选填多项）

光化性角化病（皮肤癌前病变）

发育不良痣（皮肤癌前病变）

基底细胞癌

黑色素细胞瘤

卡波式肉瘤

鳞状细胞癌

墨克尔细胞癌

表皮T细胞淋巴瘤

其它（请详述）

16. 你的家人被诊断过皮肤癌吗？（包括祖父母，父母，兄弟，姐妹，儿女，孙辈，舅舅，姑妈，表兄弟，侄子，侄女）

是

否

17. 如果前一个问题回答“是”，你的家庭成员被诊断过那种皮肤癌？（可以选填多项）

黑色素细胞瘤 基底细胞癌 鳞状细胞癌 其它皮肤癌

| 父母 |  |  |  |  |  |  |  |  |  |  |  |  |  |
| --- | --- | --- | --- | --- | --- | --- | --- | --- | --- | --- | --- | --- | --- |
| 兄弟姐妹 |  |  |  |  |  |  |  |  |  |  |  |  |  |
| 子女 |  |  |  |  |  |  |  |  |  |  |  |  |  |
| 祖父母 |  |  |  |  |  |  |  |  |  |  |  |  |  |
| 孙辈 |  |  |  |  |  |  |  |  |  |  |  |  |  |
| 舅舅/姑妈 |  |  |  |  |  |  |  |  |  |  |  |  |  |
| 侄子/侄女 |  |  |  |  |  |  |  |  |  |  |  |  |  |

18. 你做过皮肤癌的病理活检吗？

是

否

**19. 你有过以下疾病吗？（选择所有你有的疾病）**

2型糖尿病

高血压

高胆固醇

冠心病

慢性阻塞性肺病

**20. 你有过以下疾病吗？（选择所有你有的疾病）**

银屑病

佝偻病

青光眼

系统性红斑狼疮

盘状红斑狼疮

卟啉病

桥本甲状腺炎

甲亢

其它（请详述）

**21. BMI=体重（千克）/（身高（米）*身高（米）） 例如：一人身高173cm，体重76Kg，则他的BMI为76/（1.73*1.73）=25.39。 请计算你的BMI, 你的BMI更接近于哪个选项？**

18-20

21-24

25-30

31-35

>35

*** 22. 你每周运动量？**

1次每周高强度

1次每周低强度

2-4次每周高强度

2-4次每周低强度

5-7次每周高强度

5-7次每周低强度

从不运动

*** 23. 你有哪种业余爱好？（可以多选）**

艺术和手工艺

烘烤/烹饪

棒球/垒球

篮球

骑车

划船

钓鱼

园艺

高尔夫

打猎

跑步

摄影

滑雪

冲浪

游泳

网球

举重

瑜伽

其它（请描述）

24. 你有 ______________?

|  | 是 | 否， 水平偏高 | 否，水平偏低 | 我不确定 |
| --- | --- | --- | --- | --- |
| 正常胆固醇? |  |  |  |  |
| 正常甘油三酯? |  |  |  |  |
| 正常血糖? |  |  |  |  |
| 正常甲状腺水平? |  |  |  |  |
|  |  |  |  |  |

* 25. 你检测过以下哪些营养素？

|  |  | 正常 | 高 低 | 不确定或者未检测 |
| --- | --- | --- | --- | --- |
| 钙 |  |  |  |  |
| 维生素 A |  |  |  |  |
| 维生素 B |  |  |  |  |
| 维生素 C |  |  |  |  |
| 维生素 D |  |  |  |  |
| 维生素 E |  |  |  |  |
| 硒 |  |  |  |  |
| 铁 |  |  |  |  |
| 钾 |  |  |  |  |
|  |  |  |  |  |

**26. 你做过皮肤癌治疗手术吗?**

是

否

**27. 如果你前一个问题回答“是”，那么你手术的结果如何？**

肿瘤局部切除

肿瘤切除后伤残或者截肢

不确定

*** 28. 你接受过皮肤癌其它治疗吗？**

否

放射治疗

化疗

免疫治疗

生物（基因）治疗

淋巴结清除

其它（请描述）

**29. 你抽烟或者曾经抽烟吗？**

是

否

**30. 你抽哪种烟？（可以多选）**

香烟

雪茄

烟斗

水烟袋

大麻

电子烟

其它（请描述）

**31. 一天抽几包烟？**

*** 32. 抽烟年数？**

*** 33. 你用过或曾经用过咀嚼烟草吗？**

是

否

**34. 你有过因别人吸烟而被动吸烟的经历吗？**

是

否

*** 35. 你有接触过在室外吸烟，然后在2小时内又进入室内的吸烟者吗？**

是

否

36. 你现在或者曾经饮酒吗？

是

否

**37. 你每周饮用多少标准酒精量？ 一标准酒精量大约相当于12盎司（0.34公斤）啤酒， 5盎司（0.14公斤）红酒， 1.5盎司（0.04公斤）烈性酒**

*** 38. 你饮酒多少年？**

**暴饮定义为在2小时内，男性饮用5个标准酒精量，女性饮用4个标准酒精量。**

*** 39. 基于上述陈述，你是否有过暴饮？**

*** 40. 你每月有几次暴饮？**

| **从没有** | | **1次** | | **2次** | **3次** | **4次或更多** |
| --- | --- | --- | --- | --- | --- | --- |
|  | |  | |  |  |  |
|  | |  |  |  |  |  |

### 以下问题用于评估华人大众对于皮肤癌风险性和死亡率等基本认识

Top of Form

#### * 41. 你相信华人，美籍华人，或者任何有华人血统的会得皮肤癌吗？

不会得皮肤癌

得皮肤癌可能性小于白人

得皮肤癌可能性等于白人

得皮肤癌可能性大于白人

#### * 42. 你认为有色人种 （西裔，黑人，印第安人和亚裔）会得皮肤癌吗？

是

否

#### * 43. 你觉得患皮肤癌最大的外界风险因素是？

吸烟

饮酒

缺少运动

不良饮食

压力

太阳紫外线照射

环境污染

其它（请描述）

#### * 44. 什么是黑色素瘤

我不知道

一种皮肤癌

一种皮疹

不是肿瘤

其它

#### * 45. 你有阅读过政府或者私营保健机构的关于皮肤保健的最新建议吗？

是

否

我想读，但不知道如何获取这些信息

#### * 46. 防晒霜可以用于预防皮肤癌吗？

是

否

Bottom of Form

**47. 你有多担心你一生中发生皮肤癌的可能性？**

非常担心

中度担心

轻度担心

不担心

*** 48. 假如你新长了个痣或者可疑的皮肤损害，你有多大可能去看医生？**

从不

不太可能

可能

很可能

*** 49. 假如你前个问题的回答是从不或者不太可能，请解释原因？**

**50. 你的医生有问过你是否把皮肤晒成褐色或者讨论过晒成褐色的风险吗？**

是

否

*** 51. 你的医生和你讨论过你患皮肤癌的风险吗？**

是

否

*** 52. 你的医生在给你每年体检时候做全身皮肤检查吗？**

是

否

*** 53.  你的医生建议过你用防晒霜，戴宽边帽，用衣服覆盖皮肤或者戴防紫外线的眼镜吗？**

是

否

*** 54.  假如前一个问题你回答“是”，那么该医生推荐什么样的抗晒指数防晒霜？**

<15

15-30

30-50

大于 50

没有特别指定SPF水平

*** 55. 你觉得你的医生对于皮肤癌风险以及预防办法的讨论能满足你的要求吗？**

是

否

**56. 你觉得你皮肤属于哪种类型？**

1型（纯白，总会被晒伤，从不晒黑）

2型（白色，总会被晒伤，轻度晒黑）

3型（中度白，会轻度晒伤，适度晒黑）

4型（微黄，会轻度晒伤，容易晒黑）

5型（褐色，很少被晒伤，重度晒黑）

6型（黑色，从没晒伤，极度晒黑）

**57. 请选择最符合你皮肤的描述？**

|  | 全白（分数=0） | 白皙（分数=1） | 淡褐色（分数=2) | 微黄淡褐色（分数=3） | 轻度深褐色（分数=4） | 中度深褐色（分数=5） | 黑深褐色（分数=6） | 非常黑深褐色（分数=7） |
| --- | --- | --- | --- | --- | --- | --- | --- | --- |
| 你皮肤在自然情况下没被晒时的肤色？ |  |  |  |  |  |  |  |  |

* 58. 请选择最符合你皮肤的描述？

|  |  |  |  |  |  |  |  |  |
| --- | --- | --- | --- | --- | --- | --- | --- | --- |
| 如果在最大程度晒后，你皮肤的晒黑程度？ |  |  |  |  |  |  |  |  |

* 59. 你皮肤被晒黑的容易程度？

| 从没被晒黑（分数=0） | 30分钟内的阳光照射会使我皮肤晒黑（分数=1） | 30分钟到1小时的阳光照射会使我皮肤晒黑（分数=2） | 1小时到2小时的阳光照射会使我皮肤晒黑（分数=3） | 2小时到3小时的阳光照射会使我皮肤晒黑（分数=4） | 3小时到5小时的阳光照射会使我皮肤晒黑（分数=5） | 大于5小时的阳光照射会使我皮肤晒黑（分数=6） | 我总是被晒黑（分数=7） |
| --- | --- | --- | --- | --- | --- | --- | --- |
| 从没被晒黑（分数=0） |  |  |  |  |  |  |  |

* 60. 你皮肤被晒伤的容易程度？

| 我总是被晒伤（分数=0） | 30分钟内的阳光照射会使我皮肤晒伤（分数=1） | 30分钟到1小时的阳光照射会使我皮肤晒伤（分数=2） | 1小时到2小时的阳光照射会使我皮肤晒伤（分数=3） | 2小时到3小时的阳光照射会使我皮肤晒伤（分数=4） | 3小时到5小时的阳光照射会使我皮肤晒伤（分数=5） | 大于5小时的阳光照射会使我皮肤晒伤（分数=6） | 我从不被晒伤 (分数=7) |
| --- | --- | --- | --- | --- | --- | --- | --- |
| 我总是被晒伤（分数=0） |  |  |  |  |  |  |  |

*** 61. 请把前面4个问题的分值相加，总分值为以下哪个选项？**

*** 62. 你用以上标准评估你的皮肤颜色是否存在困难？**

是

否

#### 63. 你是否有___________?

|  | **是，常常** | **是，有些时候** | **是，偶尔** | **是，只在夏天** | **是，只在冬天** | **是，但很少** | **否** |
| --- | --- | --- | --- | --- | --- | --- | --- |
| **当你出门前30分钟，使用SPF>15的防晒霜并且每隔2小时重新涂抹一次？** |  |  |  |  |  |  |  |
| **使用广谱防晒霜？** |  |  |  |  |  |  |  |
| **戴有宽边帽** |  |  |  |  |  |  |  |
| **主动做日光浴晒黑？** |  |  |  |  |  |  |  |
| **室内人工晒黑（现在或者以前）？** |  |  |  |  |  |  |  |

#### * 64. 你平均一周有多少时间接受阳光照射？

#### * 65. 当你暴露在阳光下时，主要在从事什么活动（请选择所以相关选项）

工作

业余爱好

休闲活动

庭院工作

短暂暴露（比如拿信，走进汽车等）

其它（请描述）

#### 66. 你是否用防晒霜？

是

否

#### * 67. 如果你从未用过防晒霜，主要原因是什么？

不确定防晒霜的益处

没有必要使用防晒霜

使用防晒霜很麻烦

不确定如何选用防晒霜

已经使用了其它防晒方法（比如宽边帽，长袖衣服，太阳镜）

对防晒霜过敏或者担心过敏

节省开支

其它（请描述）

#### * 68. 你是否曾让你的孩子使用防晒霜？

是

否

目前还没有子女

#### * 69. 你如果给孩子使用防晒霜，你是否每隔2-3小时给孩子重新涂抹？

是

否

没有给孩子用过防晒霜

目前还没有子女

#### * 70. 当你过去曾是一个孩子的时候，你的父母让你用防晒霜吗？

是

否

#### * 71. 你是否戴宽边帽或者穿长袖服装用以避免直接阳光照射？

是

否

#### * 72. 你是否戴防止紫外线的太阳眼镜或者其它眼镜？

是

否

#### * 73. 你是否曾在外出时用伞防晒？

是

否

#### * 74. 你是否有过暴露在距离小于2英尺（60厘米）的荧光灯泡？

是

否

**感谢你参与这项调查**

非常感谢你的耐心和反馈。 你的帮助使我们获得关于皮肤癌的有用信息。我们正在从这项评估全球华人对皮肤癌的基本知识以及风险性的了解。这些信息的获取将有助于我们在现有皮肤癌防治原则基础上提出更合理的建议。

**Title of Project:** Investigating Current Knowledge and Management of Skin Cancer in the Chinese population

**Principal Investigator:** [Redacted for publication]

You are being invited to take part in a research study. Whether you take part is up to you.

Skin cancer is a global health issue. Skin cancer is becoming an epidemic in the U.S. with both melanoma patients and those with basal cell carcinoma or squamous cell carcinoma dying at a rate of one per hour. Among people of color the outcomes are far worse than that of the general populations with those who are diagnosed have poorer outcomes. This poorer outcome has been associated with delay in diagnosis due to failure of the patients and the medical community to recognize the risks of skin cancer in this population. Although the rates vary among different subpopulations of people of color, over 2 million of these individuals in the U.S. are at risk for skin cancer. Chinese has the largest population in the world. Published studies demonstrate evidence that skin cancer rates continue to rise in Chinese population. Nonetheless, compared to that of the U.S. white population, the risk factors and related research are inadequate. Thus, further research is needed for Chinese population globally, including those who reside in the U.S., China, or any other countries to assess their awareness of the risk factors and related issues of the skin cancer. As a research volunteer, you can be a patient with skin cancer or skin pre-cancer lesion, but also you can be a healthy volunteer without skin cancer. Even as a healthy volunteer without skin cancer, your knowledge of skin cancer will help us to understand any knowledge gaps in the awareness of risk factors and preventive measures of skin cancer in the Chinese population around the world.

The research purpose is to identify globally what are the gaps on knowledge of the risks factors and preventive measures of skin cancer in Chinese population in order to better address those issues in the future.

You will be asked to complete an anonymous survey that may take around 30-45 minutes to complete. You may take it at anytime and anywhere where you might have internet access that is convenient to you.

You must be 18 years of age or older to take part in this research study.

Study contact for questions about the study or to report a problem: [redacted for publication]

IRB contact about your rights in the study or to report a complaint: Research at the [redacted for publication] involving human participants is carried out under the oversight of the Institutional Review Board ([redacted for publication] IRB). This research has been reviewed and approved by the IRB. For information about the rights of people who take part in research, please contact: [redacted for publication]

1. Are you over 18 years old, able to read Chinese, and do you agree to continue with this study?
   1. Yes
   2. No
2. What is your sex?
   1. Male
   2. Female
3. What year were you born?
4. Which country were you born?
   1. China
   2. Any other Asian nations
   3. USA
   4. Canada
   5. Europe
   6. Australia
   7. Pacific islands
   8. Any other countries
5. Your country of residence?
   1. China
   2. Any other Asian nations
   3. USA
   4. Canada
   5. Europe
   6. Australia
   7. Pacific islands
   8. Any other countries
6. Are you Chinese or from Chinese descent?
   1. Yes
   2. No
7. If you answer “yes” from the previous question, please choose your ethnicity?
   1. Han
   2. Mongol
   3. Hui
   4. Tibetan
   5. Uyghur
   6. Miao
   7. Yi
   8. Zhuang
   9. Buyei
   10. Chosen
   11. Man
   12. Dong
   13. Yao
   14. Bai
   15. Tujia
   16. Hani
   17. Kasak
   18. Dai
   19. Li
   20. Any other race
8. Were you born in China? (if yes, branch to question 9; if not, branch to question 10)
   1. Yes
   2. no
9. If you were born in China, what is your ancestral home?
   1. Anhui (安徽)
   2. Beijing (北京)
   3. Chongqing (重庆)
   4. Fujian (福建)
   5. Gansu (甘肃)
   6. Guangdong (广东)
   7. Guangxi (广西)
   8. Guizhou (贵州)
   9. Hainan (海南)
   10. Hebei (河北)
   11. Heilongjiang (黑龙江)
   12. Henan (河南)
   13. Hubei (湖北)
   14. Hunan (湖南)
   15. Jiangsu (江苏)
   16. Jiangxi (江西)
   17. Jilin (吉林)
   18. Liaoning (辽宁)
   19. Inner Mongolia (内蒙古)
   20. Ningxia (宁夏)
   21. Qinghai (青海)
   22. Shaanxi (陕西)
   23. Shandong (山东)
   24. Shanxi (山西)
   25. Shanghai (上海)
   26. Sichuan (四川)
   27. Taiwan (台湾)
   28. Tianjin (天津)
   29. Tibet (西藏)
   30. Xinjiang (新疆)
   31. Yunnan (云南)
   32. Zhejiang (浙江)
   33. Hongkong (香港)
   34. Macau (澳门)
10. What is your highest level of education attained?
    1. No school education
    2. Elementary school
    3. Middle school
    4. High school
    5. College
    6. Graduate school
11. What field are you currently employed in? (If you are a student, please select "student." If you are unemployed, choose unemployed)
    1. Unemployed
    2. Agriculture
    3. Accounting
    4. Advertising
    5. Aerospace
    6. Airline
    7. Apparel and accessories
    8. Automotive
    9. Banking
    10. Biotechnology
    11. Broadcasting
    12. Call center
    13. Cargo handling
    14. Chemical
    15. Computer
    16. Construction
    17. Consumer products
    18. Cosmetics
    19. Education
    20. Electronics
    21. Energy
    22. Entertainment and leisure
    23. Financial services
    24. Food, beverage and tobacco
    25. Government
    26. Grocery
    27. Health care
    28. Internet publishing
    29. Legal
    30. Manufacturing
    31. Military
    32. Motion picture and video
    33. Music
    34. Newspaper publishers
    35. Auctions
    36. Pharmaceuticals
    37. Publishing
    38. Real estate
    39. Retail and wholesale
    40. Service
    41. Software
    42. Student
    43. Sports
    44. Technology
    45. Telecommunications
    46. Television
    47. Transportation
    48. Trucking
    49. Others (please specify)
12. If you have a second job, what field is it in? (If you are a working student, please select student; if you have no second job, select no second job)
    1. No second job
    2. Agriculture
    3. Accounting
    4. Advertising
    5. Aerospace
    6. Airline
    7. Apparel and accessories
    8. Automotive
    9. Banking
    10. Biotechnology
    11. Broadcasting
    12. Call center
    13. Cargo handling
    14. Chemical
    15. Computer
    16. Construction
    17. Consumer products
    18. Cosmetics
    19. Education
    20. Electronics
    21. Energy
    22. Entertainment and leisure
    23. Financial services
    24. Food, beverage and tobacco
    25. Government
    26. Grocery
    27. Health care
    28. Internet publishing
    29. Legal
    30. Manufacturing
    31. Military
    32. Motion picture and video
    33. Music
    34. Newspaper publishers
    35. Auctions
    36. Pharmaceuticals
    37. Publishing
    38. Real estate
    39. Retail and wholesale
    40. Service
    41. Software
    42. Student
    43. Sports
    44. Technology
    45. Telecommunications
    46. Television
    47. Transportation
    48. Trucking
    49. Others (please specify)
13. Most of your workplace is in?
    1. Indoors
    2. Outdoors
    3. Half indoors, and half outdoors
14. Have you ever been diagnosed with skin pre-cancer lesions or skin cancer?
    1. Yes
    2. No
15. If you choose “yes” in the previous question, which of the following skin pre-cancer lesions or skin cancer have you been diagnosed with? (Choose all that apply)
    1. Actinic Keratosis (pre-cancer skin lesions)
    2. Dysplastic Nevi (pre-cancer skin lesions)
    3. Basal Cell Carcinoma
    4. Squamous Cell Carcinoma
    5. Melanoma
    6. Kaposi's Sarcoma (KS)
    7. Merkel Cell Carcinoma
    8. Cutaneous T cell lymphoma (CTCL – or Mycosis Fungoides (MF))
    9. Others (please specify)
16. Has anyone in your family been diagnosed with skin cancer? (Include parents, siblings, sons, daughters, grandparents, grandsons, cousins, uncles, aunts, nieces, and nephews)
    1. Yes
    2. No
17. If you answered “yes” in previous question, which of the following skin cancer has a member of your family been diagnosed with? (Choose all that apply)

| Relationship | Melanoma | Basal cell carcinoma | Squamous cell carcinoma | Other skin cancers |
| --- | --- | --- | --- | --- |
| Parent |  |  |  |  |
| Sibling |  |  |  |  |
| Child |  |  |  |  |
| Grandparent |  |  |  |  |
| Grandchild |  |  |  |  |
| Uncle/Aunt |  |  |  |  |
| Cousin |  |  |  |  |

1. Have you ever had any skin cancer biopsy in the past?
   1. Yes
   2. No
2. Have you been diagnosed with any of the following? (Check all that apply).
   1. Type 2 Diabetes
   2. Hypertension (high blood pressure)
   3. High cholesterol
   4. Coronary Artery Disease (CAD)
   5. Chronic Obstructive Pulmonary Disease (COPD)
3. What other medical illnesses have you been diagnosed with? Check all that apply.
   1. Psoriasis
   2. Rickets
   3. Glaucoma
   4. SLE-Systemic lupus erythematosus
   5. DLE-Discoid Lupus Erythematosus
   6. Porphyria
   7. Hashimoto's Thyroiditis
   8. Graves Disease
   9. Others
4. BMI=body weight (kg)/height(m)*height(m), please calculate your BMI and fit which of the following category?
   1. 18-20
   2. 21-24
   3. 25-30
   4. 31-35
   5. >35
5. How often do you exercise?
   1. 1/week high impact
   2. 1/week low impact
   3. 2-4/week high impact
   4. 2-4/week low impact
   5. 5-7/week high impact
   6. 5-7/week low impact
   7. Never
6. Which of the following hobbies do you participate? (can choose more than one answer)
   1. Arts and crafts
   2. Baking/cooking
   3. Baseball/softball
   4. Basketball
   5. Bicycling
   6. Boating
   7. Fishing
   8. Gardening
   9. Golf
   10. Hunting
   11. Jogging / Running
   12. Photography
   13. Skiing / Snowboarding
   14. Surfing/Windsurfing
   15. Swimming
   16. Tennis
   17. Weightlifting
   18. Yoga
   19. Others (please specify)
7. Do you have…?

Yes Higher than normal Lower than normal Not sure/Not tested

1. Normal cholesterol
2. Normal triglyceride
3. Normal blood sugar level
4. Normal thyroid level
5. Have you been tested for abnormalities in any of the following nutrients?

Normal Higher than normal Lower than normal Not sure/Not tested

1. Calcium
2. Vitamin A
3. Vitamin B (complex)
4. Vitamin C
5. Vitamin D
6. Vitamin E
7. Selenium
8. Iron
9. Potassium
10. Have you ever had surgery to remove a skin cancer?
    1. Yes
    2. No
11. What was the outcome of your surgery if you answered “yes” in the previous question?
    1. Tumor (cancer) excised locally
    2. Tumor (cancer) excised resulting in deformity or amputation
    3. Uncertain
    4. No surgery for skin cancer
12. Have you had other treatments for skin cancer?
    1. No
    2. Radiation
    3. Chemotherapy
    4. Immunotherapy
    5. Biologic (genetic) therapy
    6. Lymph node dissection
    7. Others (please specify)
13. Do you or have you ever been a smoker?
    1. Yes
    2. No
14. What do you smoke? Check all that apply.
    1. Cigarettes
    2. Cigars
    3. Pipes
    4. Hookah
    5. Marijuana
    6. Electronic cigarettes
    7. Others (please specify)
15. How many packs per day do you smoke?
    1. Less than 1/4 pack
    2. 1/4 pack
    3. 1/2 pack
    4. 1 pack
    5. 2 packs
    6. 3 or more packs
16. For how many years have you smoked for?
    1. Less than 1 year
    2. 1-5 years
    3. 6-10 years
    4. 11-20 years
    5. More than 20 years
17. Do you currently, or have you ever used chewing tobacco?
    1. Yes
    2. No
18. Are you exposed to other who smoke around you?
    1. Yes
    2. No
19. Are you exposed to other who smoke outside then return to your area within 2 hours of smoking?
    1. Yes
    2. No
20. Do you drink or have you drink alcohol?
    1. Yes
    2. No
21. How many standard alcoholic drinks do you consume per week? A standard drink is considered either: 12 ounces (0.34kg) of regular beer, 5 ounces (0.14kg) of wine, or 1.5 ounces(0.04kg) of distilled spirits
    1. 0
    2. 1-2
    3. 3-4
    4. 5-7
    5. 8 or more
22. For how many years, did you or have you drank alcohol?
    1. Less than 1 year
    2. 1-5
    3. 6-10
    4. 11-15
    5. 16-20
    6. 20 or more years

Binge drinking is considered drinking more than 4 drinks in a 2 hour period as a female, or more than 5 drinks in a 2 hour period as a male.

1. Based on the statement above, have you ever participated in binge drinking?
   1. Yes
   2. No
2. How many times per month have you participated in binge drinking?
   1. Never
   2. Once
   3. Twice
   4. Three
   5. Four or more

The purpose of this page is to assess the awareness of the general population of the risk and death rate of skin cancer in Chinese.

1. Do you believe that Chinese, Chinese-American, or people of Chinese descent can get skin cancer?
   1. Never get skin cancer
   2. Less likely than Caucasians to get skin cancer
   3. Just as likely as Caucasians
   4. More likely than Caucasians
2. Do you think that people of color (Hispanic, Black, Native American, and Asian) can get skin cancer?
   1. Yes
   2. No
3. Which one do you believe is the highest risk factor for skin cancer?
   1. Smoking
   2. Alcohol
   3. Lack of exercises
   4. Lack of nutrition
   5. Stress
   6. Ultraviolet radiation from the sun
   7. Environmental pollution
   8. Others (please specify)
4. What is a melanoma?
   1. I don't know
   2. Type of skin cancer
   3. Type of skin rash
   4. Not a cancer
   5. Other
5. Do you read the latest recommendations on skin protection from any other government or private health agency source?
   1. Yes
   2. No
   3. I would like to, but it is not readily available to me
6. Is sunscreen helpful in preventing skin cancer?
   1. Yes
   2. No
7. How concerned are you that you will develop skin cancer in your lifetime?
   1. Very concerned
   2. Moderately concerned
   3. Mildly concerned
   4. Not at all concerned
8. If you developed a mole or suspicious skin lesion that was never there before, how likely would you be to go see a physician?
   1. Never
   2. Not likely
   3. Likely
   4. Very likely
9. If the answer to the previous question was never or not likely, please explain.
10. Has your physician ever asked if you tan and discussed tanning risks with you?
    1. Yes
    2. No
11. Has your physician ever talked to you about your risk of skin cancer?
    1. Yes
    2. No
12. Does your physician perform a full body skin exam on your annual checkup?
    1. Yes
    2. No
13. Does your physician advise you to wear sunscreen and/or protective clothing, wide brimmed hats UV or protective eyewear?
    1. Yes
    2. No
14. If the answer to the previous question is yes, what SPF does he/she recommend?
    1. <15
    2. 15-30
    3. 30-50
    4. Above 50
    5. No specific recommendation of which SPF level I should choose
15. Is the amount of time your physician currently discusses skin cancer risks/preventative care with you adequate for your needs and concerns?
    1. Yes
    2. No
16. How do you classify your skin type? Choose one.
    1. Type I (very fair, always burns, never tans)
    2. Type II (fair, always burns, minimal tan)
    3. Type III (medium fair, burns minimally, gradually tans)
    4. Type IV (olive, burns minimally, tans well)
    5. Type V (brown, very rarely burns, tans profusely)
    6. Type VI (dark, never burns, tans deeply)
17. Please select the most accurate descriptor for each column:
    Darkness of sun protected skin (How dark is your skin normally?)
    1. Albino (Score=0)
    2. Fair (Score=1)
    3. Tan (Score=2)
    4. Yellowish Tan (Score=3)
    5. Light Brown (Score=4)
    6. Medium Brown (Score=5)
    7. Dark Brown (Score=6)
    8. Very Dark Brown (Score=7)
18. Please select the most accurate descriptor for each column.
    Darkness of maximally tanned skin (How dark do you get if you tan?)
    1. Albino (Score=0)
    2. Fair (Score=1)
    3. Tan (Score=2)
    4. Yellowish Tan (Score=3)
    5. Light Brown (Score=4)
    6. Medium Brown (Score=5)
    7. Dark Brown (Score=6)
    8. Very Dark Brown (Score=7)
19. How easy is it for you to get a tan?
    1. I never get a tan (Score=0)
    2. I get a tan within 30 minutes of sun exposure (Score=1)
    3. I get a tan within 30 minutes and 1 hour of sun exposure (Score=2)
    4. I get a tan within 1-2 hours of sun exposure (Score=3)
    5. I get a tan within 2-3 hours of sun exposure (Score=4)
    6. I get a tan within 3-5 hours of sun exposure (Score=5)
    7. I get a tan after 5 hours of sun exposure (Score=6)
    8. I always get a tan (Score=7)
20. How easily do you get sun burned?
    1. I always get a sun burn (Score=0)
    2. I burn within 30 minutes of sun exposure (Score=1)
    3. I burn within 30 minutes and 1 hour of sun exposure (Score=2)
    4. I burn within 1-2 hours of sun exposure (Score=3)
    5. I burn within 2-3 hours of sun exposure (Score=4)
    6. I burn within 3-5 hours of sun exposure (Score=5)
    7. I burn after 5 hours of sun exposure (Score=6)
    8. I never burn (Score=7)
21. Please add up your scores for the previous 4 questions and select the total number below.
    From 0 to 28.
22. Was assessing your skin tone difficult using this scale?
    1. Yes
    2. No
23. Do you ___________?

Use SPF > 15 sunscreen applied thickly 30 minutes prior to going outside and reapply every 2 hours?

Use a broad spectrum sunscreen?

Wear a wide-brimmed hat?

Intentionally sun bathe or tan?

Indoor tan (currently or previously)?

1. Yes, frequently.
2. Yes, some days.
3. Yes, occasionally.
4. Yes, summer only.
5. Yes, winter only.
6. Yes, rarely.
7. No.
8. On average, how many hours per week do you spend in the sun?
   1. 0-5 hours
   2. 6-10 hours
   3. 11-15 hours
   4. 16-20 hours
   5. 21-25 hours
   6. 26-30 hours
   7. 31-50 hours
   8. More than 50 hours
9. The majority of hours you spend in the sun are from (please check all that apply):
   1. Work
   2. Hobbies
   3. Leisure activities
   4. Yard work
   5. Brief exposure (getting the mail, walking to the car, etc)
   6. Others (please specify)
10. Do you wear sunscreen?
    1. Yes
    2. No
11. If you do not use sunscreen, what is the main reason?
    1. Not sure of the benefits of sunscreen
    2. No need to use sunscreen
    3. Inconvenience to use sunscreen
    4. Not sure how to choose sunscreen
    5. Use other alternative sun protection methods (wide brimmed hat, long sleeve clothes, sunglasses, etc)
    6. Be allergic to sunscreen or worry about allergy
    7. Save money
    8. Others (please specify)
12. Do you instruct your children to use sunscreen?
    1. Yes
    2. No
    3. No children yet
13. If you instruct your children to use sunscreen, do you let them reapply sunscreen every 2-3 hours?
    1. Yes
    2. No
    3. Do not instruct children to use sunscreen
    4. No children yet
14. When you were a child yourself, did your parents instruct you to use sunscreen?
    1. Yes
    2. No
15. Do you wear protective clothing, such as long sleeves or wide brimmed hat to avoid direct sun exposure?
    1. Yes
    2. No
16. Do you wear protective eye wear (full spectrum UV protective sunglasses/contacts/glasses, etc.)?
    1. Yes
    2. No
17. Have you used umbrella (parasol) to protect from the sun when going outside?
    1. Yes
    2. No
18. Are you exposed to fluorescent light bulbs that are less than 2 feet (60cm) away from you?
    1. Yes
    2. No

Thank you for participating in the survey.

Your patience and insight is very much appreciated. You are helping us gather information regarding skin cancer. We are working on a research project concerning the identification and correction of gaps in knowledge, teaching, and public awareness of skin cancer risks among Chinese population globally. It is important to assess what the guidelines, beliefs of risks, and activities are currently practiced for skin cancer so we may under the current state and contrive appropriate recommendations.
