# Supplementary material for: Recommendations From a Chinese-Language Survey of Knowledge and Prevention of Skin Cancer Among Chinese Populations Internationally: Cross-sectional Questionnaire Study
Source: JMIR Dermatol. 2023 Mar 9;6:e37758. doi: 10.2196/37758 (PMC10335128; doi:10.2196/37758)
Supplement: Multimedia Appendix 2 [file derma_v6i1e37758_app2.docx]

**Select Highlights of Sun Safety and Skin Cancer-Related Resources from China**

The Central People’s Government has published some bulletins, such as an acknowledgment of the connection between artificial tanning and skin cancer [80]. The Chinese National Health and Family Planning Commission, to the best of our knowledge, has not made any official recommendations for sun protection. However, through the commission’s National Central Cancer Registry of China, they track and publish national incidence rates for skin precancerous and cancerous lesions. The National Nature and Science Foundation of China (NSFC) is one of the largest funders of dermatology research in China, including skin cancers.

For clinicians, guidelines for diagnosis and treatment of skin cancer are being developed by societies such as the Chinese Society of Clinical Oncology Melanoma Panel [64]. Non-governmental agencies, such as MediSkin, 医学界皮肤频道 (The Medical Profession: Skin Channel), 39健康网 (39 Health Network), and more provide advisories and forums for patients, researchers, and clinicians to discuss various aspects of skin cancer, including prevention. On WeChat, China’s “app for everything,” non-governmental organizations and individual physicians host official public accounts for patients, educators, and clinicians to view articles and post discussions on; examples include MediSkin (ID: pifuke_zhouxun), 医学界皮肤频道 (ID: yxj-pf), 皮肤时间 (Skin Time, ID: dxydermtoday), and 黑色素瘤患者指南 (Guide for Patients with Melanoma, ID: gh_1e43298693c9). Support groups specific to conditions may also be found, such as黑色素瘤大家谈 (Families of Patients with Melanoma Discussion, ID: gh_4e23648f56f6).

While no famous Chinese celebrities are known to have suffered or died from skin cancer who may serve to emphasize its risks, mainstream movies can be used to raise awareness. For instance, many discussions on various Chinese platforms can be found regarding malignant melanoma following the nationwide release of 《非诚勿扰2》, or “If You Are the One 2”, a 2010 feature film that showed a mole on the main character’s foot that became fatal malignant melanoma.
